# Lung Cancer Susceptibility Model Based on Age, Family History and Genetic Variants

**DOI:** 10.1371/journal.pone.0005302

**Published:** 2009-04-23

**Authors:** Robert P. Young, Raewyn J. Hopkins, Bryan A. Hay, Michael J. Epton, Graham D. Mills, Peter N. Black, Heather D. Gardner, Richard Sullivan, Gregory D. Gamble

**Affiliations:** 1 Department of Medicine, Auckland Hospital, Auckland, New Zealand; 2 Department of Oncology, Auckland Hospital, Auckland, New Zealand; 3 Department of Medicine, University of Otago, Christchurch, Canterbury, New Zealand; 4 Department of Medicine, Waikato Hospital, Hamilton, New Zealand; Stanford University, United States of America

## Abstract

**Background:**

Epidemiological and pedigree studies suggest that lung cancer results from the combined effects of age, smoking, impaired lung function and genetic factors. In a case control association study of healthy smokers and lung cancer cases, we identified genetic markers associated with either susceptibility or protection to lung cancer.

**Methodology/Principal Findings:**

We screened 157 candidate single nucleotide polymorphisms (SNP) in a discovery cohort of 439 subjects (200 controls and 239 lung cancer cases) and identified 30 SNPs associated with either the healthy smokers (protective) or lung cancer (susceptibility) phenotype. After genotyping this 30 SNP panel in a validation cohort of 491 subjects (248 controls and 207 lung cancers) and, using the same protective and susceptibility genotypes from our discovery cohort, a 20 SNP panel was selected based on replication of SNP associations in the validation cohort. Following multivariate logistic regression analyses, including the selected SNPs from runs 1 and 2, we found age and family history of lung cancer to be significantly and independently associated with lung cancer. Numeric scores were assigned to both the SNP and demographic data, and combined to form a simple algorithm of risk.

**Conclusions/Significance:**

Significant differences in the distribution of the lung cancer susceptibility score was found between normal controls and lung cancer cases, which remained after accounting for differences in lung function. Validation in other case-control and prospective cohorts are underway to further define the potential clinical utility of this model.

## Introduction

While 90% of people with lung cancer have a smoking history, only 10–15% of chronic smokers develop lung cancer suggesting factors in addition to smoking exposure are relevant [1]. Age,smoking exposure, impaired lung function and family history have been identified as independent risk factors for lung cancer [2]. Genetic factors have also been shown to play a role in determining susceptibility to lung cancer [3]. These genetic factors are believed to confer an inherent susceptibility (exaggerated or maladaptive response) to chronic inflammation from cigarette smoking [4], [5]. Consistent with many cancer models, this inflammatory stimulus in the lungs results in tissue remodeling, DNA damage and impaired cell cycle control [3]–[5]. This tissue remodeling results in impaired lung function (ie chronic obstructive pulmonary disease or COPD) that, despite affecting the minority of smokers [6], is present in 50% or more of lung cancer cases [7] and recognized as one of the most important markers of lung cancer risk [8].

Genetic predisposition to lung cancer is likely to be both polygenic and heterogeneous, conferred by a variable combination of relatively common polymorphisms with low penetrance and modest effect sizes [9], [10]. Moreover, it is likely that important smoking-gene interactions underlie lung cancer [11] as seen in other smoking-related cancers (e.g. bladder and stomach). Genetic variants associated with both COPD and lung cancer have been identified, most recently the chromosome 15q25 gene locus [12], [13]. Therefore to avoid possible confounding we suggest it is important to measure lung function in participants of case-control studies of lung cancer [13]. For both epidemiological and biostatistical reasons, spirometric screening of comparably exposed controls will increase the power of the study to identify relevant genetic variants (distinguishing low from high risk people) compared to studies where the control group is unscreened [14].

It is well known that non-genetic risk factors such as age, history of lung disease and smoking history are very important and can be combined to develop risk based tools for lung cancer susceptibility such as the Lung Cancer Assessment Tool developed by Bach (www.mskcc.org) [15]. Recently, genotype data from previously implicated prostate cancer susceptibility SNPs were combined with family history to derive risk estimates for prostate cancer [16]. In the latter study, controls were screened using prostate specific antigen and only those with normal levels were recruited as controls. This approach minimizes misclassification of controls (ie men with undiagnosed prostate cancer or at increased risk of prostate cancer). We have used a similar approach in our case control study design and analysis, and show how genetic variants previously showing small effects on lung cancer risk can be combined in an algorithm with other known risk factors to derive a risk model for lung cancer.

## Methods

### Study Population

This study was a two stage case control design conducted in 3 centers following the same recruitment protocol. Lung cancer cases of Caucasian ancestry (all 4 grandparents of Caucasian descent) were identified through hospital clinics between 2004 and 2007 as follows: >40 yrs of age, past history of smoking (minimum 15 pack years), diagnosis confirmed on histological or cytological grounds and limited to the following 4 histological subtypes- adenocarcinoma, squamous cell cancer, small cell cancer and non-small cell cancer (generally large cell or bronchoalveolar subtypes). The median time interval between diagnosis and recruitment was 3 months. Lung cancer cases underwent blood sampling for DNA extraction, an investigator administered questionnaire and spirometry using a portable spirometer (Easy-One™, ndd Medizintechnik AG, Switzerland) following American Thoracic Society (ATS) criteria. For those lung cancer cases who had already undergone surgery, pre-operative lung function performed by the hospital laboratory (using ATS criteria) was sourced from the medical records.

Control subjects were recruited from the same communities as the cases as follows: Caucasian ancestry (as defined above), aged 45–80 yrs old and had a past or current smoking history of a minimum of 15 pack years. Controls were volunteers who met the above criteria and were identified through either a community mail out or while attending community based social clubs. All smoking controls underwent blood sampling, spirometry and the same investigator administered questionnaire given to lung cancer cases. Control smokers recruited from the community that were found to have COPD, based on screening spirometry (FEV1/FVC<70% and FEV1 % predicted <80%), were analysed separately. All subjects provided informed written consent. The study was approved by the Multi-Region Ethics Committee, Wellington, New Zealand (AKX/03/08/207). The questionnaire (modified from the ATS respiratory questionnaire) included data on demographic variables such as age, gender, medical history, family history of lung disease, active and passive tobacco exposure and occupational aero-pollutant exposures.

### Selection and genotyping of single nucleotide polymorphisms

Following literature review, polymorphisms previously implicated in either COPD or lung cancer with the following attributes were selected: (a) single nucleotide polymorphisms (SNPs) in genes encoding proteins in pathways of cell-cycle control, oxidant response, apoptosis and airways inflammation and (b) SNPs that were known to have either functional effects on in vitro assays, or were non-synonymous or in regulatory regions. In a discovery cohort of 439 smokers (run 1 recruited during the years 2003–2005: 239 lung cancer cases and 200 control smokers), 157 candidate SNPs were screened (see supplementary data S1) and those where the difference in genotype frequencies between cases and controls (using recessive or co-dominant model) exceeded a 20% magnitude difference and P value <0.20 were identified as part of our model forming approach [17]. SNPs with call rates <95% after retesting, were not included in further analysis. SNPs were assigned as “protective” or susceptible when the homozygote and/or heterozygote genotype for either allele were found in excess in control smokers or lung cancer cases respectively (in a recessive or co-dominant model).

### Genotyping

Genomic DNA was extracted from whole blood samples using standard salt based methods. Purified genomic DNA was aliquoted (10 ng/ul concentration) into 96 well plates and genotyped on a Sequenom™ system (Sequenom™ Autoflex Mass Spectrometer and Samsung 24 pin nanodispenser) by the Australian Genome Research Facility (www.agrf.com.au) using sequences designed in house (available on request) and recommended amplification and separation methods (iPLEX™, www.sequenom.com) [16].

From the 157 candidate SNPs screened in our discovery cohort (see supplementary data S1), 30 SNPs met the above criteria in run 1. These 30 SNPs were genotyped in a second validation cohort of 491 smokers (run 2 recruited during the years 2006–2007: 207 lung cancer cases and 284 control smokers) recruited in the same way. For all SNP assays, again a minimum of 95% call rate was required. This second cohort of lung cancer cases and healthy control smokers were comparable to the first groups in respect to demographic factors and lung cancer characteristics (unpublished data). Based on independent replication of the associations (univariate analyses with similar OR and P values) in run 2 as observed in run 1 (ie. consistency, direction and significance of association), a final panel of the 20 most discriminatory SNPs (12 susceptibility SNPs and 8 protective SNPs from the test panel of 30) was selected (see supplementary data S1).

### Algorithm

The assignment of a protective or susceptible SNP genotype/s was made from the test cohort data (run 1) and was strictly applied to the data from run 2. For each subject, a numerical value of −1 was assigned for each of the protective genotypes present among the protective SNPs and +1 for each of the susceptible genotypes present. Where an individual did not have either the protective or susceptibility genotype for that SNP the score was 0 (ie. did not contribute to the genetic score). This approach is consistent with a recently published study in prostate cancer [16]. Weighting the presence of specific susceptible or protective genotypes according to their individual odds ratios (OR from univariate regression or point estimates from multivariate regression) did not significantly improve the discriminatory performance of the raw SNP score (unpublished data).

### Lung cancer susceptibility score

Using multivariate logistic and stepwise regression analysis from run 1, the SNPs were examined along with relevant non-genetic factors which identified age and family history of lung cancer as significant contributors to lung cancer susceptibility. Consistent with other case control studies, previously diagnosed COPD and female gender in our study were also associated with an increased risk of lung cancer (p<0.001 and p<0.01 respectively). We did not include gender in the final risk model as its importance in prospective studies is lacking [18]. We did not include COPD in the model as this was the basis of selecting our controls. Based on the multivariate analysis in run 1, a score was assigned according to age and family history and tested in run 1 and run 2 separately in a receiver operator curve analysis (ROC, see results below). These two variables have been identified in other risk assessment tools for lung cancer susceptibility [15] and improved the discriminatory power of the SNP score data alone. As smoking exposure (pack years) was a recruitment criteria for this study and comparable between cases and controls, it was not surprising to find it made little contribution to this scoring system derived from our cohorts. The lung cancer susceptibility score for the combined lung cancer cases and controls (n = 930) was plotted with (a) the frequency of lung cancer, and (b) the floating absolute risk (equivalent to odds ratio) across the combined smoker/ex-smoker cohort [19], [20].

### Statistical analysis

Patient characteristics in the cases and controls were compared by unpaired t-tests for continuous variables and chi-square test for discrete variables. Genotype and allele frequencies were checked for each SNP by Hardy Weinberg Equilibrium (HWE). Population admixture was excluded by the Population structure analysis on genotyping data from 40 unrelated SNPs [21]. Distortions in the genotype frequencies were identified between cases and controls using 2 by 3 contingency tables. Genotype data (20 SNP panel) and the most relevant non-genetic variables were combined in a stepwise fashion to assess their combined effects on discriminating low and high risk (by odds ratio and ROC) by score quintile. The frequency distribution of the optimized lung cancer susceptibility score was compared across the cases and controls. It's potential clinical utility as a risk tool was assessed using receiver-operator curve analysis.

## Results

### Demographic variables and genotyping

Characteristics of the healthy control smokers, and lung cancer cases are summarized in Table 1. The 446 lung cancer cases (run 1 = 239 and run 2 = 207) were comparable to a recently published series [22]. Given the small difference in age, the 482 healthy control smokers (run 1 = 200 run 2 = 282) were comparably exposed with respect to smoking and other aero-pollutants. The lower frequency of current smokers in the lung cancer group likely reflects co-existing COPD (higher quit rates) while longer duration of smoking in lung cancer cases reflects their older age. In a gene by smoking interaction model such as this, differences in smoking exposure are more likely to obscure effects (bias to the null) than generate effects. Consistent with the findings of others, the lung cancer cohort had higher rates of a family history of lung cancer (19% vs 9%) and history of COPD (29% vs 5%). The latter (5%) most likely reflects a clinical diagnosis of COPD, based on symptoms but not spirometry, in smokers with asthma and/or chronic bronchitis. As expected, lung function was worse in the lung cancer cohort compared to the healthy smoker controls. Testing lung function in the lung cancer cases (performed within 3 months of diagnosis, in the absence of pleural effusions and prior to surgery) allows us to test for confounding by COPD (see below).

**Table 1:** Summary of characteristics for the Lung cancer and healthy control smokers.

Parameter	Lung Cancer N = 446	Healthy control smokers N = 484	P value for differences
**Characteristics (% or mean (1SD))**			
% male	53%	60%	0.007
Age (yrs)	69 (10)	60 (10)	<0.001
Height (cm)	167(0.08)	170 (0.09)	<0.001
Weight (kg)	69 (15)	79(15)	<0.001
History of COPD	29%	5%	<0.001
**Smoking History**			
Current smoking (%)	35%	48%	<0.001
Age started (yr)	18 (4)	17 (3)	<0.001
Yrs smoked	41 (12)	35 (11)	<0.001
Pack years	41 (25)	40 (19)	0.28
Cigarettes/day	20 (10)	24 (11)	<0.001
**History of other exposures**			
In utero smoke exposure	18%	17%	ns
Mother smoked in childhood	37%	41%	0.03
Home ETS exposure as adult	79%	58%	<0.001
Work ETS exposure	86%	63%	<0.001
Work dust exposure	63%	47%	<0.001
Work fume exposure	41%	38%	0.16
Asbestos exposure	23%	16%	0.02
**Family History**			
FHx of COPD	33%	28%	0.12
FHx of lung cancer	19%	9%	<0.001
**Lung function**			
FEV1 (L)	1.86 (0.48)	2.86 (0.68)	<0.001
FEV1 % predict	73%	99%	<0.001
FEV1/FVC	64 (13)	78 (7)	<0.001
Spirometric COPD*	51%	0%	<0.001

ETS = environmental tobacco smoke.

* According to GOLD 2+ criteria.

Based on replication of association in run 1 and independently in run 2, the 20 most consistently associated SNPs were selected. The observed genotypes for the 20 SNPs in this study were in Hardy-Weinberg equilibrium (see Table 2) thereby excluding significant genotyping error. The genotype frequencies for the controls were comparable to those from the International Hapmap Project (www.hapmap.org). The development of the lung cancer susceptibility score is described in methods above and a summary of the 20 SNP panel univariate analysis is presented in Table 3. Although 6 of the top 20 SNPs do not reach traditional levels of significance they have been included in the panel because (a) in previous studies they have been shown to have functional effects (b) they have been previously associated with COPD and/or lung cancer (see discussion), (c) in combination they make a contribution to the performance of the susceptibility score (AUC for the model including only the 14 significant SNPs P≤0.05, see below), and (d) their inclusion allows for the genetic heterogeneity that exits in lung cancer case control studies.

**Table 2:** Expected genotype frequencies and Hardy Weinberg Equilibrium.

SNP #	SNP Name	rs number	Allele frequencies		HWE observed genotypes P value	Allele frequencies		Allele frequencies	
			Study total (n = 930)			Study controls (n = 484)		Hapmap –Caucasian*	
			Major	Minor		Major	Minor	Major	Minor
1	α5 nAChR	rs16969968	0.65	0.35	0.57	0.69	0.31	0.58	0.42
2	CYP 2E1	rs2031920	0.98	0.02	0.06	0.99	0.01	0.94	0.06
3	Interleukin-18	rs360721	0.70	0.30	0.77	0.68	0.32	0.70	0.32
4	Interleukin-8	rs4073	0.54	0.46	0.14	0.51	0.49	0.60	0.40
5	Interleukin 1B	rs16944	0.69	0.31	0.80	0.67	0.33	0.65	0.35
6	ITGA11	rs2306022	0.90	0.10	0.07	0.91	0.09	0.93	0.07
7	NAT 2	rs1799930	0.71	0.29	0.87	0.69	0.31	0.71	0.29
8	α1-antichymotrypsin	rs4934	0.50	0.50	0.54	0.47	0.53	0.51	0.49
9	Cerberus 1	rs10115703	0.92	0.08	0.54	0.93	0.07	0.89	0.11
10	DAT1	rs6413429	0.93	0.07	0.78	0.94	0.06	0.87	0.13
11	TNFR1	rs1139417	0.57	0.43	0.96	0.56	0.44	0.51	0.49
12	TLR9	rs5743836	0.85	0.15	0.92	0.85	0.15	0.84	0.16
13	P73 (TP73)	rs2273953	0.75	0.25	0.93	0.78	0.22	0.85	0.15
14	SOD3	rs1799895	0.99	0.01	0.99	0.98	0.02	0.97	0.03
15	ITGB3	rs2317676	0.93	0.07	0.51	0.91	0.09	0.95	0.05
16	DRD2	rs1799732	0.90	0.10	0.67	0.89	0.11	0.90	0.10
17	BCL2	rs2279115	0.51	0.49	0.60	0.53	0.47	0.57	0.43
18	XPD (ERCC2)	rs13181	0.61	0.39	0.90	0.59	0.41	0.67	0.33
19	REV1 (REV1L)	rs3087386	0.56	0.44	0.96	0.58	0.42	0.50	0.50
20	FasL (TNFSF6)	rs 763110	0.63	0.37	0.83	0.61	0.39	0.64	0.36

* allele frequencies for Caucasians from www.hapmap.org.

**Table 3:** Genotypes and results of regression analysis.

SNP *	Rs number	Genotype	LungCancer N (%)	Smoking Contr N (%)	Call rate	Univariate OR (95% CI)	*P* value	Phenotype
α5-nAChR	rs16969968	AA	68 (16%)	45 (9%)	98%	1.8 (1.2–2.7)	0.004	susceptibility
		AG/GG	361 (84%)	426 (91%)				
CYP 2E1	rs2031920	TT/TC	24 (6%)	14 (3%)	95%	2.1 (1.0–4.3)	0.03	susceptibility
		CC	379 (94%)	463 (97%)				
Interleukin-18	rs360721	CC	237 (54%)	208 (45%)	96%	1.4 (1.1–1.9)	0.009	susceptibility
		CG/GG	201 (46%)	250 (55%)				
Interleukin-8	rs4073	TT	129 (31%)	109 (23%)	96%	1.5 (1.1–2.1)	0.005	susceptibility
		AT/AA	284 (69%)	367 (77%)				
Interleukin 1B	rs16944	GG	215 (49%)	212 (44%)	99%	1.2 (0.9–1.6)	0.14	susceptibility
		AA/AG	224 (51%)	269 (56%)				
ITGA11	rs2306022	AA	14 (3%)	6 (1%)	98%	2.6 (0.9–7.6)	0.04	susceptibility
		GA/GG	422 (97%)	470 (99%)				
N–acetylcysteine transferase 2	rs1799930	GG	239 (56%)	222 (47%)	97%	1.4 (1.1–1.9)	0.006	susceptibility
		AA/AG	189 (44%)	253 (53%)				
α1-antichymotrypsin	rs4934	GG	123 (28%)	96 (20%)	98%	1.6 (1.2–2.2)	0.004	susceptibility
		AG/AA	312 (72%)	383 (80%)				
Cerberus 1	rs10115703	AA/AG	71 (16%)	59 (12%)	97%	1.4 (0.9–2.0)	0.10	susceptibility
		GG	363 (84%)	413 (88%)				
DAT1	rs6413429	GT/TT	64 (15%)	50 (10%)	98%	1.5 (1.0–2.3)	0.04	susceptibility
		GG	367 (85%)	431 (90%)				
TNFR1 (TNFRSF1A)	rs1139417	AA	148 (36%)	142 (30%)	96%	1.3 (1.0–1.8)	0.05	susceptibility
		AG/GG	258 (64%)	329 (70%)				
TLR9	rs5743836	CC	12 (3%)	6 (1%)	96%	2.2 (0.8–6.6)	0.12	susceptibility
		CT/TT	419 (97%)	455 (99%)				
P73 (TP73)	rs2273953	CC	219 (52%)	292 (62%)	96%	0.65 (0.49–0.85)	0.001	protective
		TC/TT	206 (48%)	178 (38%)				
SOD3	rs1799895	GG/GC	4 (1%)	15 (3%)	96%	0.28 (0.10–0.90)	0.02	protective
		CC	425 (99%)	451 (97%)				
ITGB3	rs2317676	GG/GA	44 (10%)	77 (16%)	98%	0.59 (0.39–0.89)	0.008	protective
		AA	391 (90%)	403 (84%)				
DRD2	rs1799732	CDel/Del.Del	70 (16%)	107 (22%)	98%	0.68 (0.48–0.96)	0.02	protective
		CC	359 (84%)	372 (78%)				
BCL2	rs2279115	AA	103 (24%)	145 (31%)	97%	0.71 (0.53–0.97)	0.03	protective
		AC/CC	328 (76%)	330 69%)				
XPD (ERCC2)	rs13181	GG	60 (14%)	81 (18%)	96%	0.74 (0.51–1.10)	0.11	protective
		GT/TT	376 (86%)	377 (82%)				
REV1 (REV1L)	rs3087386	CC	128 (29%)	163 (34%)	98%	0.79 (0.59–1.10)	0.10	protective
		TC/TT	310 (71%)	312 (66%)				
FasL (TNFSF6)	rs 763110	TT	53 (12%)	78 (16%)	98%	0.72 (0.49–1.10)	0.09	protective
		TC/CC	379 (88%)	403 (84%)				

* (OMIM nomenclature).

### Risk model development

In a multivariate logistic regression analysis that included the selected SNPs (individually), age (>60 yrs), family history of lung cancer (first degree relative), gender and history of COPD were found to be independently associated with lung cancer susceptibility in run 1, run 2 and combined. For the combined data set, OR for the susceptibility and protective SNPs ranged between 1.1–3.2 and 0.20–0.80 respectively (the combined SNP score is independently related to lung cancer, P<0.001). The OR for age>60 yrs and family history of lung cancer were 3.5 (2.5–4.9, p<0.001) and 2.5 (1.6–4.0, p<0.001) respectively (total AUC = 0.75 where SNPs were included individually while adjusting for the non-genetic variables). Based on these findings, and those from previously published studies [3], [6], [7], we assigned scores to non-genetic variables as follows; +4 for those aged >60 yrs old and +3 for those with a family history of lung cancer. Such an approach is consistent with existing risk scores [15], [16] and places the SNP data in appropriate clinical context [15]. Gender and diagnosed COPD were not included in this risk model for the reasons described above.

### Model performance

**Figure 1:**
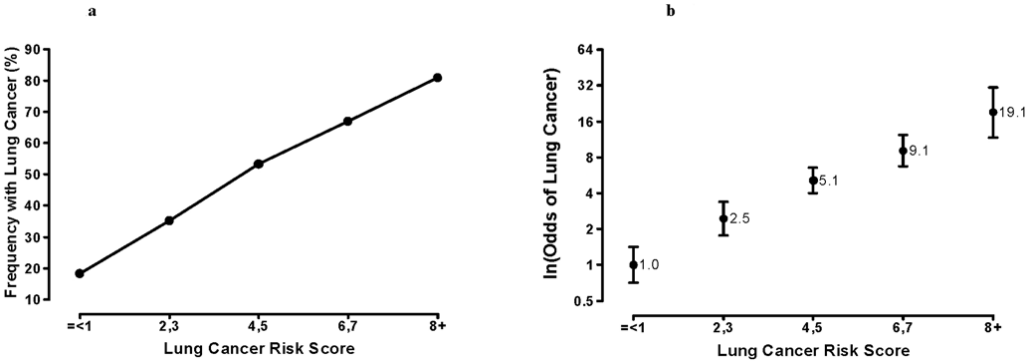
1a. Frequency of lung cancer according to the lung cancer susceptibility (risk) score. 1b. Odds ratio of lung cancer according to the lung cancer susceptibility (risk) score.

**Figure 2:**
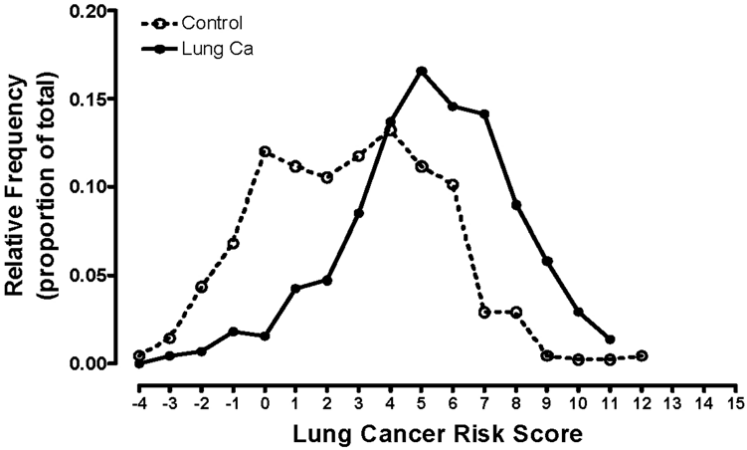
Frequency distribution of the lung cancer susceptibility (risk) score in cases and controls.

In the combined 20 SNP model, the lung cancer susceptibility score was compared with frequency of lung cancer and a linear relationship was found across the lung cancer susceptibility scores ≤1 to 8+ with lung cancer frequency spanning 18% to 81% (figure 1a). The magnitude of this effect was also examined using the floating absolute risk [19], [20] plotted on a log scale (equivalent to an Odds ratio, OR), which references the lowest frequency group as OR = 1 (referent group, lung cancer score ≤1) and compares each lung cancer score relative to the referent group (Figure 1b). The OR spanned from 1 to 19.1 across the lung cancer scores when subjects were grouped approximately as quintiles (p<0.001). The lung cancer susceptibility score for lung cancer cases and controls shows a bimodal distribution on frequency distribution (Figure 2) indicating potential utility as a screening test of risk [23].

### Model sensitivity analysis

**Figure 3:**
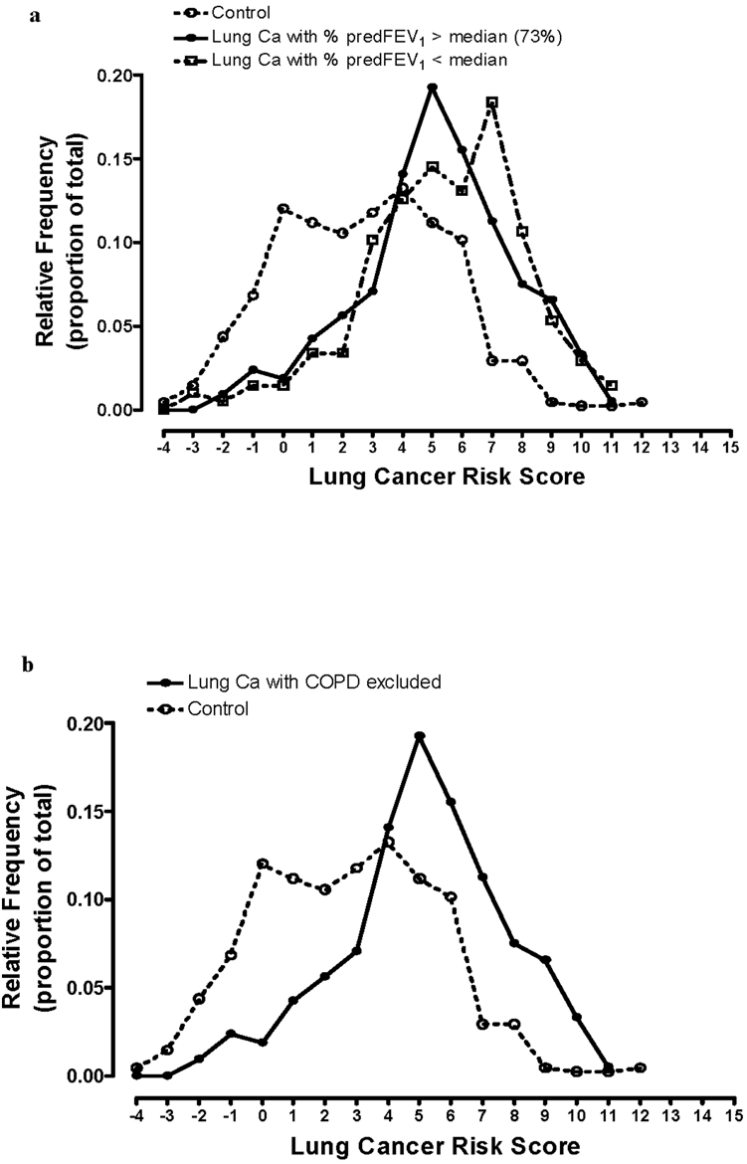
3a Frequency distribution of the lung cancer score among controls and lung cancer cases divided according to low (COPD) and normal lung function. 3b Frequency distribution of the lung cancer score among controls and lung cancer cases with normal lung function (COPD excluded).

To correct for the small differences in age, smoking status and gender mix between cases and controls, a subgroup (sensitivity) analysis was done (a) limited to those over 60 years of age (age weighting equally applied to all) and (b) where mean age, pack years and gender were closely matched between cases and controls (n = 450: 72 vs 69 yrs, 45 vs 43 pack years and 70% vs 70% male respectively). A linear increase in OR across quintiles of the lung cancer susceptibility score (range 1–28, p<0.01) remained evident with confidence intervals consistent (ie. overlapping) with those derived using the full data set (figure 1b). The potential confounding effect of COPD was also examined by (a) comparing the distribution of the lung cancer susceptibility score in lung cancer cases according to spirometric criteria (% predicted FEV1, Figure 3a) and (b) excluding lung cancer cases with co-existing COPD (based on previously described spirometric criteria n = 227, Figure 3b). The distribution of the scores among cancer cases sub-grouped according to lung function or COPD are not different to the total lung cancer cohort (Figures 3a and 3b) and exclude significant confounding by COPD.

### ROC analysis

In a receiver operator curved analysis (n = 930) of the combined 20 SNP model, we found the area under the curve (AUC or C statistic) for run 1, run 2 and run 1+2 was 0.82, 0.75 and 0.77 respectively. The AUC in the total cohort for the 20 SNP panel, age, and family history of lung cancer on their own were 0.68, 0.70 and 0.55 respectively. When “genetic factors” only are utilised in the risk model (SNPs+FHx of lung cancer), as seen in the Prostate cancer study [16], the OR spans 1–10 across quintiles and the AUC = 0.70 (with no contribution from age). On stepwise analysis, age and the SNP panel make the greatest contribution to the AUC (SNPs = 0.68, age+SNPs = 0.76 and age+SNPs+FHx = 0.77). When the SNP panel is limited to the 14 significant SNPs, the AUC for the SNPs alone is 0.66 and when combined with age and family history is 0.75. When gender was included in the 20 SNP combined model the AUC was not improved. When past history of COPD was also added to the combined model (scoring +4 based on multivariate regression), the AUC increased to 0.79. As stated above, when age and pack years were stringently matched and possible confounding by COPD analysed, there was no difference in our findings.

## Discussion

Using a candidate gene approach in a two stage selection process a panel of protective and susceptibility SNPs were identified that individually confer only small effects on risk of lung cancer (OR ranging from 0.3 to 2.6). This is very much in keeping with the experience from case control association studies to date [11], [12], [16], [24]. Consistent with existing risk models, relevant factors were combined using an algorithm (in this study including SNP data) to derive a susceptibility score on a simple linear scale. This study design, and the algorithmic approach that underlies this lung cancer susceptibility score, is comparable to a recent study in prostate cancer. Moreover, it takes into account important epidemiological observations relevant to genetic predisposition to lung cancer. First, that although smoking exposure is essentially a pre-requisite to getting lung cancer, increasing age and poor lung function have important independent effects on lung cancer susceptibility. Second, the genetic factors underlying lung cancer risk are likely to be both polygenic and heterogeneous, conferred by a variable combination of genetic variants (i.e. SNPs with low penetrance and small effect sizes). Third, genetic factors may confer either a protective [24] or susceptibility [13] phenotype to lung cancer. Fourth, the potential confounding effect of COPD [13] has been accounted for in the model. Here we report a 20 SNP panel which combined with family history [16] define risk (OR) across quintiles ranging 1–10 with an AUC of 0.70. A risk tool with greater clinical utility can be derived by including age to identify those at greatest susceptibility to lung cancer (OR ranging 1–19 and AUC = 0.77).

This study sought to minimize false positive results in a number of ways. The most important of these was to internally validate the SNP associations using a two stage design with an initial discovery cohort (run 1) to identify SNPs of potential interest. Only these SNPs were tested in a second (validation) cohort of cases and controls (run 2) and using univariate analysis from the two runs independently to select the SNPs based on replication. Second, population stratification was excluded and third, the presence of genotyping error was minimized through HWE analysis and by the exclusion of SNPs with <95% call rate (fails on genotyping is invariably genotype specific, thus generating false positive associations). With respect to possible confounding, in a sensitivity analysis where lung cancer cases and healthy smoking controls were matched for smoking exposure (pack years), age, gender and presence of COPD, the performance of the lung cancer score was not reduced.

Weaknesses in this study include the modest size of the cohorts, borderline significance of some SNPs in the absence of correction, cross-sectional design and recruitment limited to Caucasians with a minimum 15 pack years. Furthermore, we chose to recruit smokers with essentially normal lung function as controls to improve power [14] and best represent those least susceptible to the adverse effects of smoking (COPD and lung cancer) but most representative of smokers in general who maintain normal lung function [6]. For this reason, COPD was not included in the model although it is an important risk factor and added to the score's utility in a post-hoc analysis. A further limitation of the study is that although the cases and controls were arguably representative, not all variables were precisely matched in the initial analysis (eg age, gender and smoking patterns). It should be noted that although precise matching of all demographic variables reduces the potential for confounding, it also potentially obscures important effects of variables in a risk model. Although only 14 of the 20 SNPs reached traditional levels of significance in the combined cohorts, and the addition of the remaining six SNPs only contributed modestly to the model, this was a two stage design where replication of associations (in this and other studies) and biological plausibility [23]–[42] were the basis of SNP selection. Further studies will need to be done to further validate this SNP panel and risk model in unselected populations.

In this study a candidate gene (i.e. hypothesis driven) approach was used to identify potentially functional SNPs associated with the development of both COPD and lung cancer. Although the SNPs identified in this study may only reflect linkage disequilibrium with functional variants nearby, these SNPs are likely to have functional effects and involvement directly with susceptibility to lung cancer. The 20 SNP panel consists of genetic variants known to encode proteins underlying important pathways implicated in lung carcinogenesis, specifically; metabolism of smoking-derived carcinogens (N-Acetyl Transferase 2 and Cytochrome P450 2E1) [25], [26], inflammatory cytokines (Interleukins 1, 8 and 18, Tissue necrosis factor alpha1 receptor, Toll-like receptor 9) [27]–[30], smoking addiction (dopamine D2 receptor and Dopamine transporter 1) [31], [32], anti-oxidant response to smoking (α1 anti-chymotrypsin and extracellular superoxide dismutase) [24], [33], cell cycle control, DNA repair and apoptosis (Xeroderma Pigmentosum complementary group D, p73, Bcl-2, FasL, Cerb1 and REV1) [34]–[39] and integrins implicated in apoptosis [40]–[42]. One of the SNPs (α5 nAChR) has recently been associated with both lung cancer and COPD in candidate gene [13] and genome wide association studies [43], [44]. This receptor appears to de directly related to nicotine effects on airway inflammation [45]. As can be seen, the SNP panel (Table III) is made up of a variety of SNPs from genes implicated in many inter-related pathways. Twelve of these SNPs have been associated with lung cancer in other cohorts. It is likely other SNPs from as yet unidentified genes will be identified in the future. To assess further the utility of the lung cancer susceptibility score, a prospective study is in progress. To date the lung cancer cases (n = 43) have the same mean and distribution as the lung cancer cases reported in this study (unpublished data). Further case control and functional studies will be needed to further explore the role of these SNPs in lung cancer susceptibility.

The authors propose that clinical utility of genotype data requires that many SNPs are analyzed and their effects combined with other epidemiological factors of relevance [16]. The algorithm approach used in this study assumes a simple additive model comparable to that recently published in Prostate cancer [16] and involves minimal assumptions (not hierarchical or Path analysis based). The patient's score can be compared with the scores in smokers with least susceptibility to lung cancer (lowest quintiles) in a simple linear fashion. Such an approach is comparable to the risk tools developed by others [15], [16]. The potential clinical utility of the lung cancer susceptibility score was assessed by receiver operator curve analysis. This showed the c statistic to be 0.77 and, at a cut off of ≥3, an estimated sensitivity of 89% and corresponding specificity of 45%. These findings are comparable to the ROC performance of the Framingham score (c statistic = 0.74). The c statistic for the 20 SNP panel on its own was 0.68 (and 0.70 when combined with family history) indicating its utility in the current cohort. There is evidence, although limited, that genetic testing may positively alter the behavior of smokers in the context of smoking cessation (increase intent and possibly improve quit rate [46], [47]) or by lowering smoking prevalence [48]. Although further validation studies are required, this study suggests that genetic data may be combined with other risk variables from smokers or ex-smokers to identify individuals most susceptible to developing lung cancer. Further studies are planned in larger cohorts of unselected cases and controls.

## Supporting Information

Supplementary Data S1 (0.03 MB DOC)
