# Supplementary material for: Lung Cancer Susceptibility Model Based on Age, Family History and Genetic Variants
Source: PLoS One. 2009 Apr 23;4(4):e5302. doi: 10.1371/journal.pone.0005302 (PMC2668761; doi:10.1371/journal.pone.0005302)
Supplement: Supplementary Data S1 — (0.03 MB DOC) [file pone.0005302.s001.doc]

**PLOS One (Supplementary material)**

**Lung cancer susceptibility model based on age, family history and genetic variants.**

Robert P.Young*^1,5^, Raewyn J.Hopkins^1^, Bryan A. Hay^1^, Michael J. Epton^3^, Graham D. Mills^4^, Peter N. Black^1^, Heather D. Gardner^1^, Richard Sullivan^2^ & Gregory D. Gamble^1^.

**Screening SNPs for run 1 (OMIM nomenclature) – SNPs identified in the test cohort (run 1, n=30) are in bold and those selected from the validation cohort (run 2, n=20) are bold and underlined.**

AACT **(rs4934),** PI (**rs17580**, Z allele), PARP1 (rs1136410), APEX (rs3136820), ARG1 (rs2781667, rs2781659, rs2246012), ARG2 (rs3742880), ATM (rs1800056, rs664143), BARD1 (rs1129804), BCL2 (rs1564483, rs3943258, **rs2279115**), BRCA1 (rs799917), BRCA2 (**rs144848**), BUB1B (rs1801376), C5R1 (rs4804049), CASP9 (rs4645978, rs4645981), CCND1 (**rs603965**), CHEK2 (rs17879961), CHRNA5 (**rs16969968**), COMT (rs4680), CSF3 (rs2227316), PTGS2 (rs5275), CTGF (CTGF−447CG), CCNH (rs2266690), CYP17 (rs743572), CYP1A1 (**rs1048943**), CYP1A2 (rs2470890, rs762551), CYP1B1 (rs1056836), CYP2A13 (rs8192789), CYP2A6 (rs1801272, rs28399433), CYP2E (**rs2031920**, rs6413432, rs3813867), CYP3A43 (**c74delA**), DAT1 (**rs6413429**), DEFB1 (rs2738047), DNMT3B (rs2424913), DR4 (rs4871857), DRD2 (**rs1799732**, rs1076560, rs6276), DRD4 (rs1800955), PTGER2 (rs708494), ELA2 (rs3826946), ELA2 (rs2007647), ERCC1 (rs3212986, rs11615), ERCC2 (rs1799793, **rs13181**), ERCC5 (rs17655), ERCC6 (rs2228526), ESR1 (rs2077647, rs1801132), TNFRSF6 (rs1800682), TNFSF6 (**rs763110**), FUS2 (rs2269432), GADD45 (rs532446), CSF3 (rs25645), GSTA1 (rs1051775), GSTM3 (rs7483), GSTP1 (rs947894, rs1138272), HAVCR2 (rs10515746), HSPA1L (rs2227956), IFNG (rs2430561), IFNGR2 (rs1059293), IGF2R (rs8191754), IGFBP3 (rs2854744), IL12A (rs582054, rs568408), IL13 (rs20541, rs1800925), IL16 (rs4778889), IL18 (rs549908, **rs360721**), IL1A (rs17561, rs1800587), IL1B (**rs16944**, rs1143627), IL6 (rs1800795, rs1800796), IL8 (**rs4073**), ITGB3 (**rs2317676**), EMT (rs451494), LIG4 (rs2232641, rs1805389), LRMP (rs7969931), MBL2 (rs1800450), MDM2 (rs2279744), ABCB1 (rs1045642), EPHX1 (rs2234922, rs1051740), MGMT (rs12917), MLH3 (rs175080), MMP1 (rs1799750), MMP12 (rs652438, rs2276109), MMP2 (rs243865), MMP3 (rs3025058), MMP7 (rs17880821, rs17881472), MMP9 (rs3918242), MPO (rs2333227), MTHFR (rs1801133), MUC5AC (rs17859812), NAT1 (rs15561), NAT2 (**rs1799930**, rs1208, rs1799929, rs1799931), NBS1 (rs1805794), NOS3 (**rs1799983**, rs2070744), NQO1 (rs1800566), OGG1 (**rs1052133**), CDKN1A (rs1801270), CDKN1B (rs2066827), CDC2 (Lys106Arg), TP53 (**rs1042522**), TP73 (**rs2273953**), PCNA (rs3626), PDCD5 (rs1862214), POLI (rs8305), PPARG (rs1801282), PTEN (rs3830675), RAD51 (rs1801320), RAD52 (rs11226), RB1 (rs2854344), REV1L (**rs3087386**), SOD2 (rs1799725), SOD3 (**rs1799895**), STAT3 (rs957971), SULT1A1 (rs9282861), TGFB1 (rs1982073), TLR9 (**rs5743836**), TNFRSF1A (rs4149584), UGB (rs3741240), XPA (rs1800975), XPC (rs2228001), XRCC1 (**rs25487**), XRCC2 (rs3218536), XRCC3 (rs861539), XRCC4 (**rs1056503**) PI7 (rs6747096), PI7 (rs3795879), TNF (rs1800629), TNF (rs1800610), PTGIS (rs5580), ITGA11 (**rs2306022**), CER1 (**rs10115703**), TNFRSF1A (**rs1139417**).
